# Changes in Body Composition and Its Relationship to Performance in Elite Female Track and Field Athletes Transitioning to the Senior Division

**DOI:** 10.3390/sports8090115

**Published:** 2020-08-20

**Authors:** Yuka Tsukahara, Suguru Torii, Fumihiro Yamasawa, Jun Iwamoto, Takanobu Otsuka, Hideyuki Goto, Torao Kusakabe, Hideo Matsumoto, Takao Akama

**Affiliations:** 1 Waseda Institute for Sport Sciences, Waseda University, Tokorozawa 359-1192, Japan; 2 Institute for Integrated Sports Medicine, Keio University, Tokyo 160-0016, Japan; 3 Faculty of Sport Sciences, Waseda University, Tokorozawa 359-1192, Japan; shunto@y.waseda.jp (S.T.); takao-akama@waseda.jp (T.A.); 4 Marubeni Health Promotion Center, Tokyo 103-6060, Japan; Yamasawa-F@marubeni.com; 5 Bone and Joint Disease Center, Keiyu Orthopaedic Hospital, Tatebayashi 374-0013, Japan; jiwamotoexmed@yahoo.co.jp; 6 School of Education, Tokai Gakuen University, Nagoya 468-8514, Japan; otsuka-t@tokaigakuen-u.ac.jp; 7 Department of Health and Fitness, Faculty of Wellness, Shigakkan University, Obu 474-8651, Japan; hide-g@fj8.so-net.ne.jp; 8 Department of Orthopedic Surgery, Japanese Red-Cross Kyoto Daini Hospital, Kyoto 602-8026, Japan; torao-k@helen.ocn.ne.jp; 9 Public Interest Incorporated Foundation, Japan Sports Medicine Foundation, Tokyo 150-0012, Japan; keiohideo@jcom.zaq.ne.jp

**Keywords:** body composition, elite female athlete, percentage fat mass, percentage lean mass, track and field

## Abstract

Many elite female athletes struggle to maintain performance while transitioning from high school to university-level (senior) sports. This study explores factors of body composition that influenced performance in elite junior female track and field athletes transitioning to the senior division. Forty-two elite female track and field athletes, ranked among the top 100 in Japan, were enrolled in this study. Whole-body mode dual-energy X-ray absorptiometry scans were performed during the post-season of 2016 and 2017. Athletes’ performances were assessed using the International Association of Athletics Federation scoring system. Relationships between changes in performance and those in body composition were investigated. There were significant negative correlations between changes in performance and fat mass (FM), and percentage FM (FM%). This was seen in total body and lower extremities, and not in the trunk and upper extremities. In addition, there was a positive correlation between changes in performance and percentage lean mass (LM%). However, there were no correlations between changes in performance and LM and total mass. Elite female track and field athletes transitioning to senior division should decrease their FM and FM% and increase LM%, to sustain or improve performance. It is also more important to monitor changes in body composition than body mass.

## 1. Introduction

Maintaining performance is a crucial issue for elite athletes aiming to continue their sports activities. Elite junior track and field athletes are competing at a higher level year by year in both national and international events, and in fact, in 2019, four outdoor events in women have set a world U20 record [1,2]. However, being an elite junior athlete does not always guide them to be a successful elite senior athlete [3], and the database of The Inter-University Athletics Union of Japan and All Japan High School Athletic Federation shows fewer female athletes in college than in high school [4]. From 2010 through 2014, there were 11–19 female sprinters in the final year of high school in Japan who ran 400 m in less than 56 s but only 2–7 in the first year of college [5,6]. As a matter of fact, Hollings et al. has reported that from 1986 to 2006, 74% of those who competed at World Junior Championships were not able to represent their country at the senior level [7]. These findings suggest that many elite junior track and field female athletes are often unable to maintain their performance during the transition from high school to college. Thus, even though many junior athletes struggle to maintain or enhance performance in the senior division, there is no scientifically evidenced solution for this problem.

Factors of body composition and its correlation to performances have been investigated in various types of sports [8,9,10,11]. However, studies focused on female athletes are limited [12]. Women tend to accumulate subcutaneous fat from increased sex hormones after puberty and during early adulthood [13,14,15]. A relationship between the longitudinal changes in lean body mass and the performance of young elite female soccer players has been reported [16]. Furthermore, Legaz et al. reported that body fat measured by skinfold thickness harmed the performances of track and field athletes [17,18]. However, the relationship of change in body composition and performance are not fully understood, especially among high-performing elite track and field females transitioning to the senior division. Thus, the purpose of this study was to explore factors of body composition that would influence performance in elite female track and field athletes transitioning to the senior division in track and field.

Because fat accumulation occurs during early adulthood, and fat mass has been reported to have a negative influence on performance, we sought the relationship of performances with body fat and other body composition factors. Since sprinters and jumpers mainly rely on lower extremities, we hypothesized that the body composition of the lower extremities has a stronger relationship to their performances than that of other regions.

## 2. Materials and Methods

### 2.1. Subjects

The study participants were female track and field athletes who ranked among the top 100 in Japan, including those who participated in the International Association of Athletics Federation (IAAF) world junior or youth championships. All participants were aged 17 or 18 years at the school year of 2016. The athletes were mostly recruited from the Kanto, Kansai and Tokai regions and were examined at a designated hospital. Athletes who specialized in one of the following four categories were enrolled: Sprinters (100 m, 200 m, 400 m, 100 m Hurdle, and 400 m Hurdle), middle distance runners (800 m and 1500 m), jumpers (long jump and triple jump), and heptathlon athletes. We decided to exclude long-distance runners from this study, since, in Japan, the “ekiden (long-distance relay running race mostly on roads)” season begins after track season and does not have an off-season. Throwers, high jumpers, and pole vault athletes were also excluded, since these events are more of technique sports, and throwers tend to have a substantially different body composition to other track and field athletes [19]. We also excluded athletes who did not participate in any event and did not have a season record. All athletes were examined by a sports medicine physician beforehand and were excluded if they had any active injuries during the measurement. Written informed consent was obtained from both the subjects and their legal guardians after being informed of the benefits and risks of the investigation. This study was carried out at the authors’ affiliated institutions and performed according to the guidelines of the Declaration of Helsinki. The project was approved by the Ethics Review Procedures Concerning Research with Human Subjects Group of the authors’ affiliated institutions (approval number 2015–323).

### 2.2. Design

Longitudinal data were obtained at post-season in 2016 (T-1) and 2017 (T-2) during a one-year period. We defined post-season as October–December, since track and field season is roughly from April through October in Japan. We used the records from September–October as their performance each year.

### 2.3. Season Performance

On a questionnaire, athletes wrote their best season performance, and we scored the records using IAAF scoring tables of athletics 2017 [20], which is a method to compare equivalent performances across different events in athletics. If an athlete competed in several events, we used the highest score.

### 2.4. Height, Weight, and Body Composition

Height was measured without shoes to the nearest 0.1 cm using a stadiometer with the athlete standing erect. Body weight was measured before training, and at least 2 h after from their last meal. Body composition was assessed by whole-body mode dual-energy X-ray absorptiometry (DXA) using a device from Hologic Delphi A (Hologic, Bedford, MA, USA). After calibration, the subjects, wearing a t-shirt without objects that interfere with the test, lay on the table with their shoulders slightly abducted and elbows slightly flexed, so that their forearms were parallel to the body axis. Their fingers were kept extended without spreading with their palms on the table. A polystyrene block, in the shape of an isosceles triangle with the vertex angle of 30 degrees, was placed between the feet, which were bound to the block, so that the fibulae were not be hidden by the tibiae. Total mass (TM), lean mass (LM), fat mass (FM), bone mineral content (BMC), percentage lean mass (LM%), percentage fat mass (FM%), and percentage bone mass (BM%) were measured in the upper extremities (UE), trunk, lower extremities (LE), and total body. We used TM of the whole body for body weight, since there was little, if any, difference between the two. The same technician performed the scans at each site.

### 2.5. Training Schedules

On a questionnaire, athletes answered their training hours per day on both weekdays and weekends.

### 2.6. Statistical Analysis

The data were analyzed with the Statistical Package for Social Sciences Statistics 22 (IBM Japan Ltd., Tokyo, Japan). The results are listed as means ± standard deviation (SD). The Shapiro-Wilk test was applied to assess distribution. Pearson correlation analyses were conducted to explore the association between performance and body composition. Continuous data were compared using the independent t-test. *p*-values less than 0.05 were considered significant.

## 3. Results

Total of 42 athletes proceeded to analysis. Characteristics of the subjects, including season performance and training hours per day are listed in Table 1. Their season performances and training hours per day were not significantly different between the two timelines.

### Association between Performance and Body Composition

- Total body

**Figure 1:**
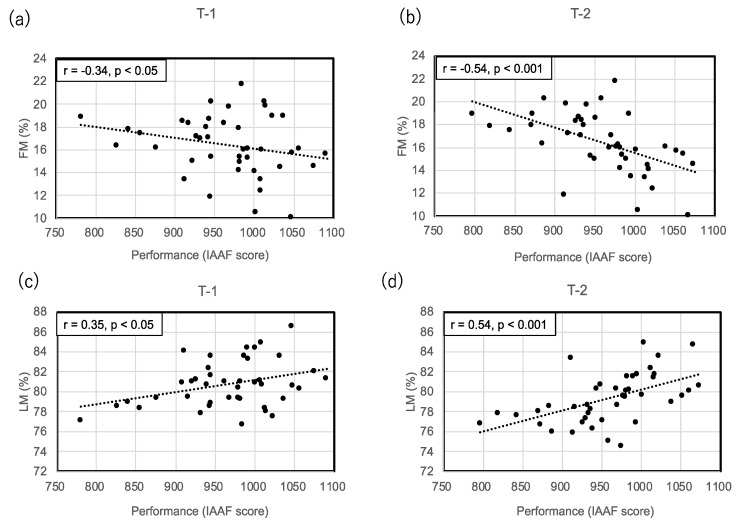
Performance (IAAF score) and its correlation with (**a**) FM% in T-1; (**b**) FM% in T-2; (**c**) LM% in T-1; (**d**) LM% in T-2. T-1: post season of 2016; T-2: post-season of 2017. LM%: percentage lean mass; FM%: percentage fat mass.

**Figure 2:**
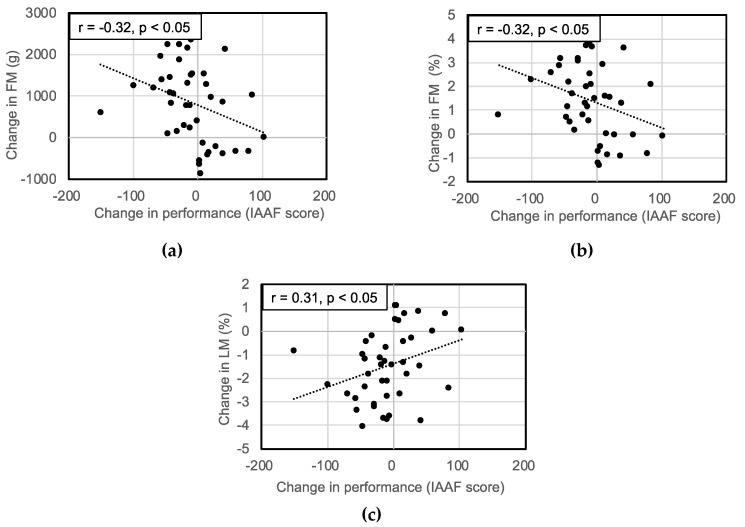
Correlations between changes in performance (IAAF score) and (**a**) FM; (**b**) FM%; (**c**) LM% in total body.FM: fat mass; FM%: percentage fat mass; LM%: percentage lean mass.

TM, FM, BMC, FM% and BM% increased and LM% decreased significantly after a year. Body composition variables are listed in Table 2. Moderate correlations were found between IAAF score and FM% (r = −0.34, *p* < 0.05) and LM% (r = 0.35, *p* < 0.05) in T-1, and FM% (r = −0.54, *p* < 0.001) and LM% (r = 0.54, *p* < 0.001) in T-2 (Figure 1). There were also significant negative correlations between changes in performance and FM (r = −0.32, *p* < 0.05) and FM% (r = −0.32, *p* < 0.05). Furthermore, there were positive correlation between changes in performance and LM% (r = 0.31, *p* < 0.05). However, there were no correlations between changes in performance and LM and TM (Figure 2).

- Upper extremities

FM% increased, while LM and LM% decreased significantly, after a year (Table 2). There were no correlations between changes in performance and any of the factors of body composition.

- Trunk

TM, FM, BMC and FM% increased, while LM% decreased significantly, after a year (Table 2). There were no correlations between changes in performance and any of the factors of body composition.

- Lower extremities

**Figure 3:**
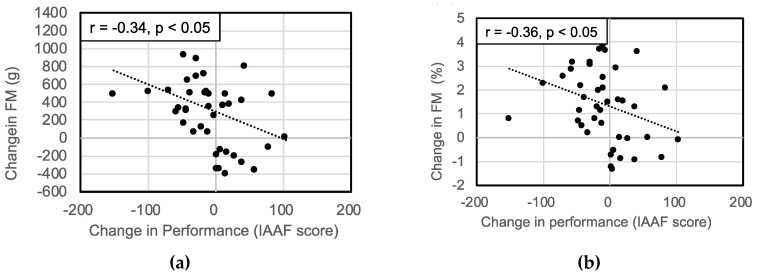

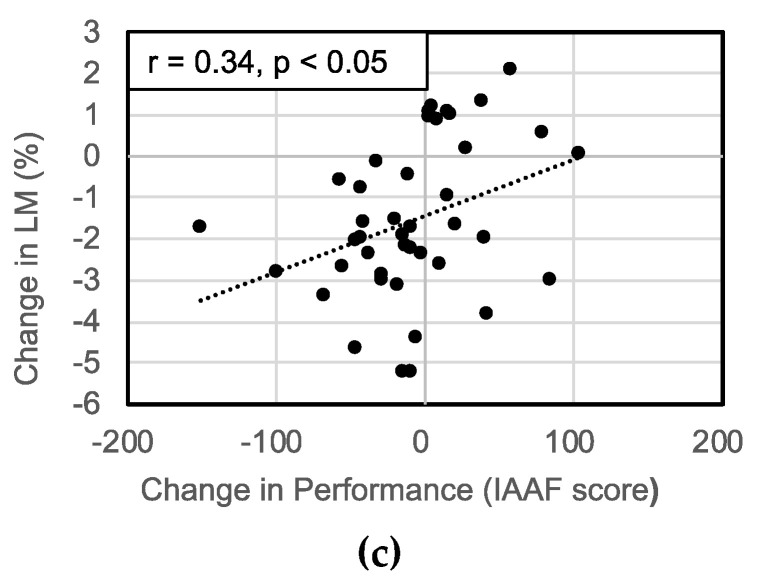
Correlations between changes in performance and (**a**) FM; (**b**) FM%; (**c**) LM% in lower extremities.FM: fat mass; FM%: percentage fat mass; LM%: percentage lean mass.

FM, BMC and FM% increased, while LM and LM% decreased significantly, after a year (Table 2). Significant negative correlations have been found between changes in performance and FM (r = −0.34, *p* < 0.05) and FM% (r = −0.36, *p* < 0.05). Furthermore, there were positive correlation between changes in performance and LM% (r = 0.34, *p* < 0.05; Figure 3).

## 4. Discussion

We investigated the relationships between changes in performance and body composition over a period of one year among elite female track and field athletes; such a longitudinal study has not been done to the best of our knowledge. In both T-1 and T-2, performance was associated with FM% and LM%, and as a whole, although TM increased significantly after a year, FM and FM% increased more significantly (Table 2). In addition, since there were no significant changes in LM after a year, whereas LM% decreased significantly. We surmised that the decrease in LM% was mostly due to an increase in FM rather than the decrease in LM. Legaz and Eston reported that a decrease in skinfold thickness, used to represent subcutaneous fat thickness, correlated with performance improvement in Spanish athletes of sprints, middle distance, and jumps whose IAAF scores were around 1000, which are close to those of our subjects [18]. They also reported that the sum of skinfold measurements of the legs correlated negatively with performance level in Ethiopian long-distance runners [21], and a similar study demonstrated a correlation with skinfold and sprint test in elite female basketball players [22]. While they measured subcutaneous fat thickness, and we measured FM, both studies indicate a negative correlation between performance and FM. In addition, there was a negative relationship between the fat mass of the trunk and peak speed and lean mass of the arm and final time in cross-country skiers [23,24]. In this study, changes in FM and FM% were associated with changes in performance in total body and lower extremities, and not in upper extremities and trunk. This is not surprising, since our subjects were sprinters and jumpers, and their performance, mainly relies on lower extremities [25].

Although coaches reported using body weight as an important index for athletes’ physiques [26], since there was no correlation between change in TM and performance in our study, we surmise that a change in body weight itself is not correlated with sports performance, whereas FM and FM% does. Given the fact that elite female track and field athletes gain fat during the transition to the senior division and could affect their performance, thus monitoring body composition and attempting to decrease FM% are important considerations. However, we should be aware not to lower FM too much for the female athlete triad and relative energy deficiency in sport, which is typically seen in long-distance runners to occur but fortunately, our subjects were not so low as to be of concern [27,28]. Though Kang et al. reported that LM in females in general reaches plateau at the age of 16–17 years [29], which was consistent with our findings that LM of the whole body did not change significantly after a year. However, LM in lower extremities decreased significantly after a year in our athletes. Kooreman et al. demonstrated that high-intensity training had led to significantly higher LM compared to low-intensity training, indicating that, if properly trained, late teenage female athletes can increase LM [30]. Thus, the decrease of LM in lower extremities could be due to the decrease in training intensity which was not measured in our research, and future studies are needed. In addition, according to a study on swimmers, LM was significantly larger in elite swimmers than recreational swimmers even though height, body weight, body fat percentage and body mass index were not significantly different between the two groups, meaning that LM% could have a relationship to performance [31].

Several limitations of this study should be noted. First, we were only able to obtain data for two school years, and the numbers of subjects were not sufficient to perform multiple regression analysis, which could have been more suitable in our study, although 42 is quite a large number when dealing with elite-level athletes. In addition, even though the actual number of subjects participated in this study was much larger than 42, the majority of subjects did not participate in events in some seasons—some did not perform that season, and therefore, were not analyzed. Investigating the cause of the fact that many subjects could not participate in some seasons would be warranted. Accordingly, we plan to continue this longitudinal study for a longer duration and with a larger cohort. Second, previous studies have indicated a positive correlation between dietary fat intake and an increase in body fat over one year in adolescent females [32], which may have happened to our subjects as well. Thus, it would have been ideal to have obtained information about their dietary intake. Last, despite the fact that it is challenging especially for sprinters and jumpers, it is necessary to measure their training load and seek if there were any changes during this timeline. Further studies are needed to address these issues.

Track and field is a sport that allows easy evaluation of athlete performance. Even though the use of DXA scanning to assess body composition is increasing [33], many coaches still consider body weight as an important factor in assessing performance. However, in our study, this was not the case; rather, FM, FM% and LM% were important factors that correlated with performance. Given the fact that most athletes gained fat during the transition from the junior to senior division and were unable to enhance their performance levels, regular measurement of body composition and attempts to maintain it appear important, as opposed to an excessive focus on body weight, even though measuring body weight is much easier and cheaper than measuring body composition. Although we used DXA scanning, which must be conducted by certified technicians, other ways to measure body composition are available [34].

## 5. Conclusions

This one-year study examined the influence of body composition on performance in elite female track and field athletes transitioning to senior. The following findings were obtained: FM and FM% increased significantly, especially in the total body and lower extremities after a year, and changes in these were associated with performance. Thus, it is important for elite female track and field athletes not to increase FM and FM%, and increase LM%, in order to maintain or improve their performance. It is recommended to assess body composition from time to time in addition to measuring their body weight.

## Figures and Tables

**Table 1:** Characteristics of the subjects.

	T-1	T-2	Changes from T-1 to T-2	*t* Value	*p* Value
Age (years)	17.7 ± 0.6	18.6 ± 0.6	1.0 ± 0.0	129.01	<0.001
Height (cm)	162.8 ± 5.2	163.0 ± 5.2	0.2 ± 0.6	1.59	0.12
Body weight (kg)	53.6 ± 5.3	54.2 ± 5.0	0.6 ± 1.6	2.36	0.02
BMI (kg/m^2^)	20.2 ± 1.6	20.4 ± 1.6	0.2 ± 0.6	2.09	0.04
IAAF Score	967.1 ± 66.8	958.6 ± 64.3	−8.4 ± 47.1	1.16	0.25
Training hours on weekdays (hours/day)	2.8 ± 0.6	2.8 ± 0.6	−0.1 ± 0.8	0.53	0.60
Training hours on weekends (hours/day)	3.5 ± 0.9	3.6 ± 0.7	0.0 ± 0.8	0.36	0.72

Results are expressed as mean ± standard deviation. T-1, post-season of 2016; T-2, post-season of 2017. BMI, body mass index; IAAF, International Association of Athletics Federation.

**Table 2:** Body composition (total body) during T-1 and T-2.

**Whole Body**	**T-1**	**T-2**	**Changes from T-1 to T-2**	***t*** **Value**	***p*** **Value**
TM (kg)	53.6 ± 5.3	54.2 ± 5.0	0.6 ± 1.6	2.36	0.02
LM (kg)	43.2 ± 3.7	42.9 ± 3.5	−0.3 ± 1.3	1.46	0.15
FM (kg)	8.1 ± 1.9	9.0 ± 2.0	0.8 ± 0.9	5.76	<0.001
BMC (g)	2239.8 ± 249.7	2293.0 ± 231.1	53.2 ± 76.3	4.52	<0.001
LM (%)	80.8 ± 2.3	79.3 ± 2.5	−1.5 ± 1.5	6.22	<0.001
FM (%)	15.0 ± 2.4	16.4 ± 2.6	1.4 ± 1.5	5.86	<0.001
BM (%)	4.2 ± 0.4	4.2 ± 0.4	0.1 ± 0.1	2.47	0.02
**Upper Extremities**	**T-1**	**T-2**	**Changes from T-1 to T-2**	***t*** **Value**	***p*** **Value**
TM (kg)	5.5 ± 0.6	5.4 ± 0.5	−0.1 ± 0.4	1.37	0.18
LM (kg)	4.3 ± 0.4	4.2 ± 0.4	−0.1 ± 0.2	3.09	<0.01
FM (kg)	0.9 ± 0.3	1.0 ± 0.2	0.0 ± 0.2	1.64	0.11
BMC (g)	266.1 ± 31.6	264.7 ± 26.5	−1.4 ± 15.4	0.61	0.55
LM (%)	78.8 ± 3.8	77.5 ± 3.5	−1.2 ± 2.6	3.01	<0.01
FM (%)	16.4 ± 4.0	18.3 ± 3.9	1.9 ± 2.8	4.38	<0.001
BM (%)	4.9 ± 0.4	4.9 ± 0.4	0.0 ± 0.2	1.29	0.21
**Trunk**	**T-1**	**T-2**	**Changes from T-1 to T-2**	***t*** **Value**	***p*** **Value**
TM (kg)	23.7 ± 2.5	24.2 ± 2.4	0.5 ± 0.8	3.97	<0.001
LM (kg)	20.3 ± 1.9	20.3 ± 1.8	0.0 ± 0.7	0.07	0.95
FM (kg)	2.7 ± 0.7	3.2 ± 0.9	0.5 ± 0.5	6.21	<0.001
BMC (g)	610.2 ± 78.8	629.3 ± 79.7	19.1 ± 20.8	5.96	<0.001
LM (%)	86.0 ± 2.3	84.2 ± 2.8	−1.8 ± 1.9	6.03	<0.001
FM (%)	11.5 ± 2.4	13.2 ± 2.9	1.7 ± 1.9	5.87	<0.001
BM (%)	2.6 ± 0.3	2.6 ± 0.3	0.0 ± 0.1	1.69	0.10
**Lower Extremities**	**T-1**	**T-2**	**Changes from T-1 to T-2**	***t*** **Value**	***p*** **Value**
TM (kg)	19.8 ± 2.2	19.9 ± 2.2	0.1 ± 0.7	1.14	0.26
LM (kg)	15.4 ± 1.5	15.2 ± 1.5	−0.2 ± 0.6	2.42	0.02
FM (kg)	3.6 ± 0.9	3.9 ± 0.9	0.3 ± 0.4	4.91	<0.001
BMC (g)	858.9 ± 99.6	869.0 ± 96.9	10.2 ± 30.6	2.15	0.04
LM (%)	77.8 ± 3.1	76.2 ± 3.2	−1.6 ± 1.9	5.37	<0.001
FM (%)	17.8 ± 3.1	19.4 ± 3.2	1.5 ± 1.9	5.19	<0.001
BM (%)	4.4 ± 0.4	4.4 ± 0.4	0.0 ± 0.2	0.98	0.33

Results are expressed as mean ± standard deviation. T-1, post-season of 2016; T-2, post-season of 2017. TM, total mass; LM, lean mass; FM, fat mass; BMC, bone mineral content; LM%, percentage lean mass; FM%, percentage fat mass; BM%, percentage bone mass.
